# Effect of *Nigella sativa* on immune response in treadmill exercised rat

**DOI:** 10.1186/1472-6882-14-437

**Published:** 2014-11-07

**Authors:** Zahra Gholamnezhad, Mohammad Hossein Boskabady, Mahmoud Hosseini

**Affiliations:** Pharmacological Research Center of Medicinal Plants and Department of Physiology, School of Medicine, Mashhad University of Medical Sciences, Mashhad, Iran; Neurogenic Inflammation Research Center and Department of Physiology, School of Medicine, Mashhad University of Medical Sciences, Mashhad, Iran; Neurocognitive Research Centre, School of Medicine, Mashhad University of Medical Sciences, Mashhad, Iran

**Keywords:** *Nigella sativa*, Cytokine, Th1/Th2 balance, Moderate exercise, Overtraining exercise

## Abstract

**Background:**

In the present study the effect of *Nigella sativa* (*N. sativa*) ethanolic extract on cytokine profile in control, moderate and overtrained heavy exercised rat was examined.

**Methods:**

Male Wistar rats were randomly divided into control sedentary (C), moderate trained (MT), (V = 20 m/min, 30 min/day, 6 days a week, for 8 weeks), overtrained (OT) (V = 25 m/min, 60 min/day, 6 days a week, for 11 weeks), control sedentary + *N. sativa* (NC), moderate trained + *N. sativa* (NM) and overtrained + *N. sativa* (NO). Immediately and 24 h after the last bout of exercise blood samples were obtained. The serum concentrations of TNFα, IL-6, IL-10, IL-4 and IFNγ were measured by ELISA method.

**Results:**

Immediately after exercise the following findings were observed; IL-6, IL-10 and TNFα concentration increased in OT and NC groups but Just IL-6 in MT groups compared with control (*P*< 0.05-*P*< 0.001). Serum level of IL-4 decreased in MT and NC (*P*< 0.05-*P*< 0.001) but IFNγ increased (*P*< 0.05) just in MT group vs control. In addition, circulatory levels of TNFα, IL-6, IL-10 and IL-4 were higher in OT and NM groups but the IFNγ concentration was lower in the OT group than the MT group (*P*< 0.05-*P*< 0.01). The IFN-γ/IL4 ratio was significantly increased in MT and NC (*P*< 0.05*-P*< 0.01) while it decreased in OT group. There were not statistical differences in TNFα, IL-6, and IFNγ levels between different time intervals after exercise in all groups.

**Conclusions:**

Chronic administration of *N. sativa* may change pro and anti-inflammatory cytokines profiles. Also it may act as a balancing factor on Th1/Th2 lymphocytes in different exercise loads and act as an inhibitory factor on Th2 phenotype in control animals.

## Background

Herbal medicines which are derived from plants and plant extracts had been traditionally used for health improvement and scientists in recent decades are interested in understanding their mechanism of action and identifying their main constituents. Many studies have been done to show the beneficial therapeutic effects of herbal medicines, including anti-oxidant, anti-inflammatory, anti-cancer, anti-microbial, and immunomodulatory (immunostimulant or immunosuppressant) [1]. There are a large number of such plants like *Corcus sativus*, *Allium sativum*, *Thymus vulgaris*, *Zataria multiflora*, and *Nigella sativa* (*N. sativa*).

Among them black seed or *N. sativa* that belongs to the Ranunculacea family has a rich historical and religious background [2]. It is native to southwest Asia but it has been cultivated and used in different parts of the world as a spice, food additive, as well as herbal medicine for a wide range of illnesses, including bronchial asthma, headache, toothache, nasal congestion, infections, obesity, back pain, hypertension, diarrhea, gastrointestinal problems and many types of cancer [3, 4]. Many studies have shown in vivo and in vitro anti-oxidant, anti-inflammatory, anti-microbial, antitumor, and immunomodulatory properties of *N. sativa* and its ingredients. The protective effect of *N. sativa* against toxicity of lead [5], carbon tetrachloride [6], cisplatin [7] and other toxins on liver, kidney and hematological parameter had also been shown. The anti-inflammatory effects of *N. sativa* and its constituents have also been reported in several diseases like experimental allergic encephalomyelitis, colitis, arthritis [1], sensitized animals [8, 9], asthmatic patients [10–12] and chemical war victims [11].

T cell cytokines have a pivotal role in the promotion of immune responses against invading pathogens [13]. There are two distinct cytokine producing T cell subtypes: CD4^+^ T helper and CD8^+^ T cytotoxic which T helpers have been appointed to type 1 and type 2 T lymphocytes based on their profile of cytokine production [14]. Type 1 lymphocytes are essential for the cell-mediated immune and defense against intracellular pathogen by producing interferon *γ* (IFN*γ*), interleukin 2 (IL-2) and tumor necrosis factor-β cytokines. Whereas, type 2 lymphocytes produce cytokines including IL-4, IL-5, IL-6, IL-10, and IL-13 and are responsible for defense against extracellular pathogens by the development of humoral immunity [15, 16]. These two classes of cytokines have cross-regulatory signaling, for example IL-4 and IL-10 secretion causes the inhibition of Th1-type immune responses by down-regulating of macrophage-derived IL-12 production. But also, IFN*γ* changes the balance of Th1/Th2 by suppressing the Th2-type immune responses [17].

Different kinds of exercise (as physical stressors) may affect immune parameters based on the nature, intensity and time delay between exercise bouts. It has been reported that moderate or intermittent exercise training is associated with anti-inflammatory and beneficial effects on immunological functions like immune polarization toward Th1 lymphocyte [18–20]. This adaptive effect may mediate the health-beneficial effect and play an important role in protecting against chronic non-communicable diseases which are associated with low-grade inflammation [21]. However, with overtraining, which is still a poorly understood process, the homeostatic balance involving a wide range of hormonal, metabolic, and immunologic factors is altered [22]. Many evidences suggested that overtrained athletes exhibit an immune shift towards the Th2 phenotype. Our previous study was also showed that prolonged and overtraining exercise causes numerous changes in immunity that possibly reflects physiological stress and immune suppression [23]. So overtrained athletes may be at increased risk of upper respiratory tract infection (URTI) during periods of sever exercise and for a few weeks after race events [24]. Although many studies had evaluated the effect of sports nutrition on post exercises immunomodulation in Olympic and marathon race athletes, the study of forced sever exercise and overtraining syndrome in human has clear ethical limits and there are not any recommended treatment for it in the literature [18, 20, 25].

Regarding many recommendations for prophylactic usage of the *N. sativa* for healing fatigue and body strengthening, we examined the effect of its ethanolic extract in control, moderate and overtrained heavy exercised rats. So, the aim of the present study was to evaluate the tonic effect of *N. sativa* on the immune system by evaluating the serum concentrations of TNFα, IL-6, IL-10, IL-4 and IFNγ cytokines, immediately and 24 h after the last bout of exercise.

## Methods

### Preparation of extract

*N. sativa* seeds were purchased from a local herb store. The plant was identified by botanists in the herbarium of Ferdowsi University of Mashhad, and the specimen number of the plant is 293-0303-1. The hydroethanolic extract was prepared using a maceration method as follows: 200 g of chopped *N. sativa* seeds were mixed with 800 ml of 50% ethanol for 72 h at 40°C. The solutions after filtration were dried by rotary evaporation at 40°C and the solvent (ethanol + water) was totally removed by rotary evaporation under reduced pressure. This process was repeated three times during the study. The dried extract was then solved in drinking water to yield the described concentration. Total amount of *N. sativa* seed was purchased before starting the study and were stored at 4°C in dark closed container. So plant extract preparation situation and materials were the same during the study period.

### Animals

Forty adult (6-8 week old) male Wistar rats, (Animal House of School of Medicine, Mashhad University of Medical Sciences, Mashhad, IR Iran) weighing 150-200 g were used. Animals were housed under environmentally controlled conditions (12 h light/dark cycle; 22-24°C) food and water was available *ad libitum* throughout the experiment. Animals were allowed to adjust to new condition for two weeks. The protocols used conformed to guidelines of the conduct animal studies, were approved by the committee on the ethics of animal experiments in Mashhad University of Medical Sciences.

### Training protocol

A motorized treadmill with 4 individual lanes was used. A shock grid at the back of the treadmill provided a mild shock (0.5 mA, 1 HZ) if the rat’s pace went below the treadmill rate during familiarizing period which is recommended by previous study [25, 26] and it was shown that it is not interfere with the measurements. The animals were undertaken a familiarizing period for one week before the beginning of the experiments. They were placed on the treadmill 5 days for 10 min/day at a speed of 12 m/min at 0% degree inclination. Then they were scored 1-5 depending on running quality and rats that run voluntarily with mean rating 3 or higher (n = 24) from those refused (n = 6) had been separated and were chosen for the study. This procedure was used to exclude possible different levels of stress between animals [25].

Thirty six rats were randomly divided into two main equal treated and untreated groups, which each one divided into three subgroups including: control sedentary (C), moderate trained (MT) and overtrained (OT). Treated animals received 200 mg/kg/day of *N. sativa* extract, which was added to their drinking water fresh daily during the training time period. Therefore, the six groups of animal were studied including: control sedentary (C), moderate trained (MT), overtrained (OT), control sedentary + *N. sativa* (NC), moderate trained + *N. sativa* (NM) and overtrained + *N. sativa* (NO).

The animals of the both control groups (treated and none treated) were handled and placed on the treadmill to experience the stress of treadmill environment. Exercised groups undertook a progressive load training 6 days a week to enhance cardiorespiratory fitness and a 5 minute warm up and cool down were done each session. Moderate trained groups underwent 8-week exercise at a speed of 15 m/min for 20 min, 6 days/week but the intensity of exercise was increased to 20 m/min for 30 min at the onset of the second week [27]. Overtrained groups were submitted into 3 phase program. In the first 4 weeks (phase I) training speed increased from 15 to 25 m/min and training time from 20 min to 60 min. In the second 4 weeks (phase II) training load was maintained constant. During last 3 weeks (phase III) running intensity and training duration remained unchanged but recovery time between training sessions was reduced (from 24 h to 4, 3 and 2 h). In this study a standard training protocol for overtraining with an imbalance period between exercise bouts and rest was used. The using protocol caused a decline in performance as shown in our previous study (submited to Plant Foods Hum Nutr) and other studies [25, 26].

### Sample collection

At the end of the study, animals were anesthetized with diethyl ether and peripheral blood was collected from the retro-orbital sinus in the control group, immediately and 24 h after the last session of exercise in MT, OT, NC, NM and NO groups. A light anesthesia with ether was used in this study for all groups. Therefore, if the anesthesia procedure adopted influence the results, it would influence in all groups similarly. After allowing blood to clot on ice, serum samples were separated by centrifuging at 3000 rpm for 10 min. Serum was collected and stored at -20°C for cytokine analysis. According to literature recommendations [28], a standard method for blood collection and processing was used to avoid any hemolysis and platelet activation. Groups cytokines changes in different times are variable (increment and decrement could be seen in cytokine pattern), so it may not be due to any microscopic hemolysis.

### Cytokine assays

Cytokine determination was performed with commercially platinum ELISA kits (Bender Med system, Austria) according to manufacturers’ instruction. They were carefully checked for specify, sensitivity and reliability. Serum concentration of IL-4, IL-6, IL-10, TNFα and IFNγ were measured using rat ELISA kits: BMS628, BMS625, BMS629, BMS622 and BMS621 respectively. The absorbance was measured using a spectrophotometer and a microplate reader (Biotek, USA) and concentration of each cytokine was calculated by comparison curve established in the same measurement using prism 5 Graphpad. Each cytokine assay was performed in duplicate each time.

### Statistical analysis

The results were presented as means ± SEM. Group-data comparisons (more than two groups) were performed using one way analysis of variance (ANOVA) with Tukey-Kramer post-test. Group-data comparisons (two groups) were performed using unpaired “t” test. The impact of time on each group data was evaluated by paired “t” test. Significance was accepted at *P*< 0.05.

## Results

### The effect of exercise intensity on serum concentration of IL-6, TNFα, IL-10, IL-4 and IFNγ

**Figure 1:**
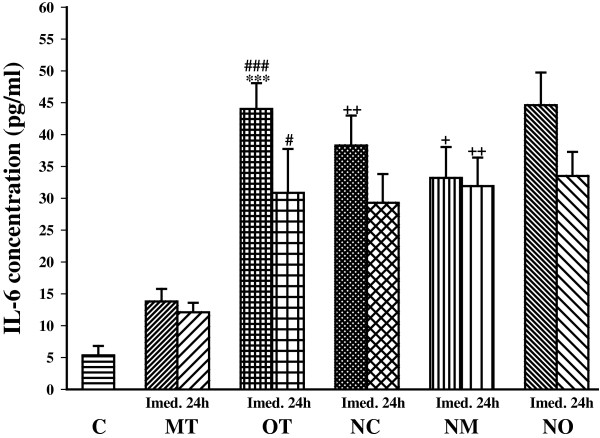
**The serum concentrations of IL-6 in non treated groups including; control sedentary rats (C), moderate trained (MT), overtrained (OT), and treated groups with** ***N. sativa*** **: control sedentary +** ***N. sativa*** **(NC), moderate trained +** ***N. sativa*** **(NM), overtrained +** ***N. sativa*** **(NO), (for each group, n = 6).** ***; *P*< 0.001, compared to control group. ^#^; *P*< 0.05, ^###^; *P*< 0.001, comparison between overtrained and moderate exercise.^+^; *P*< 0.05, ^++^; *P*< 0.01, comparison between treated and non treated groups.

Immediately after the last bout of exercise IL-6 concentration increased non significantly after moderate exercise (MT group) compared to control animals. But IL-6 concentration was significantly increased in overtraining (OT group) (*P*< 0.01) compared with control. In addition, IL-6 values were higher in the OT group than the MT group (*P*< 0.001) which remained elevated after 24 h (*P*< 0.05) (Figure 1).

**Figure 2:**
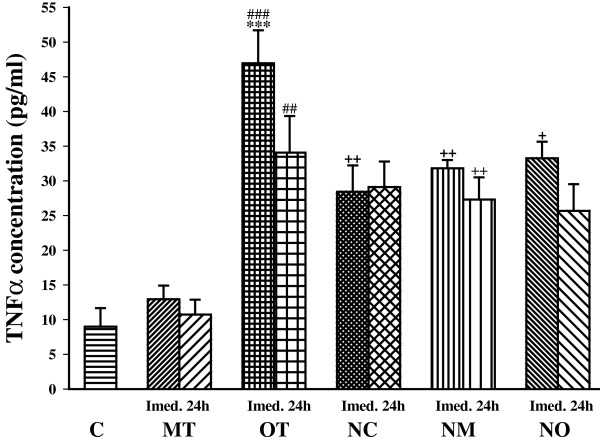
**The serum concentrations of TNFα in non treated groups including; control sedentary rats (C), moderate trained (MT), overtrained (OT), and treated groups with** ***N. sativa*** **: control sedentary +** ***N. sativa*** **(NC), moderate trained +** ***N. sativa*** **(NM), overtrained +** ***N. sativa*** **(NO), (for each group, n = 6).** ***; *P*< 0.001, compared to control group. ^##^; *P*< 0.01, ^###^; *P*< 0.001, comparison between overtrained and moderate exercise.^+^; *P*< 0.05, ^++^; *P*< 0.01, comparison between treated and non treated groups.

TNFα concentration was also significantly increased immediately after the last bout of exercise in OT (*P*< 0.001) compared with control. In addition, TNFα concentration was higher in OT than MT immediately (*P*< 0.001) and 24 h after (*P*< 0.01) the last bout of exercise (Figure 2).

**Figure 3:**
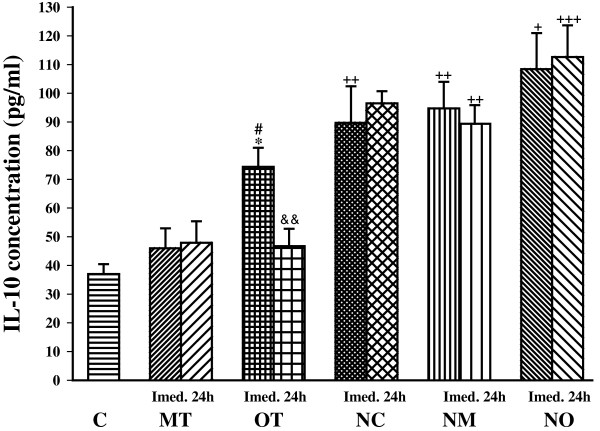
**The serum concentrations of IL-10 in non treated groups including; control sedentary rats (C), moderate trained (MT), overtrained (OT), and treated groups with** ***N. sativa*** **: control sedentary +** ***N. sativa*** **(NC), moderate trained +** ***N. sativa*** **(NM), overtrained +** ***N. sativa*** **(NO), (for each group, n = 6).** *; *P*< 0.05, compared to control group. ^#^; *P*< 0.05, comparison between overtrained and moderate exercise.^+^; *P*< 0.05, ^++^; *P*< 0.01, ^+++^; *P*< 0.001, comparison between treated and non treated groups. ^&&^; *P*< 0.01, comparison between immediately and 24 h after last bout of exercise.

Immediately after the last bout of exercise IL-10 concentration was significantly increased in OT (*P*< 0.05) compared with the control. In addition, IL-10 concentration was higher in OT than MT (*P*< 0.05) immediately after the last bout of exercise (Figure 3).

**Figure 4:**
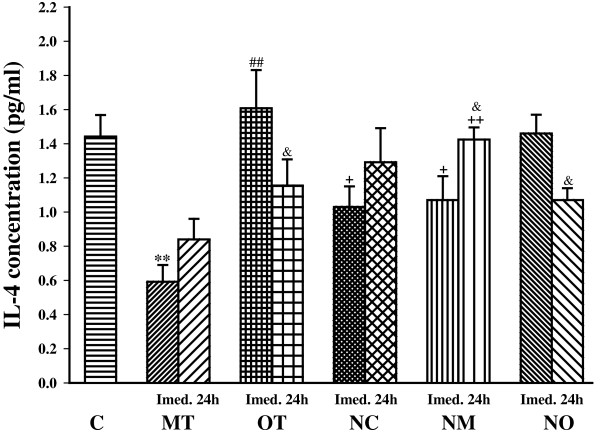
**The serum concentrations of IL-4 in non treated groups including; control sedentary rats (C), moderate trained (MT), overtrained (OT), and treated groups with** ***N. sativa*** **: control sedentary +** ***N. sativa*** **(NC), moderate trained +** ***N. sativa*** **(NM), overtrained +** ***N. sativa*** **(NO), (for each group, n = 6).** **; *P*< 0.01, compared to control group. ^##^; *P*< 0.01, comparison between overtrained and moderate exercise.^+^; *P*< 0.05, ^++^; *P*< 0.01, comparison between treated and non treated groups. ^&^; *P*< 0.05, comparison between immediately and 24 h after last bout of exercise.

Immediately after the last bout of exercise serum level of IL-4 was significantly decreased in MT (*P*< 0.01) compared with control. IL-4 concentration was significantly higher in the OT group than the MT group (*P*< 0.01) immediately after the last bout of exercise (Figure 4).

**Figure 5:**
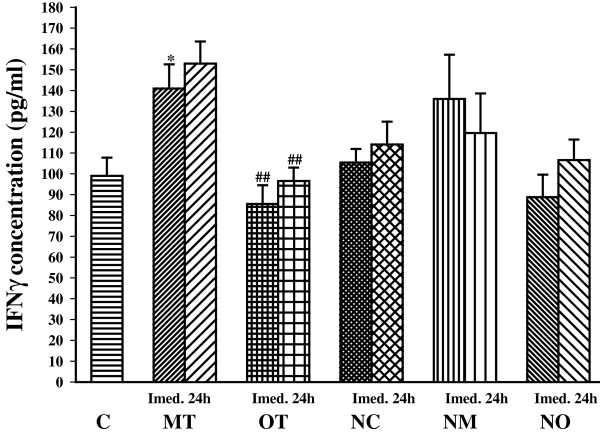
**The serum concentrations of IFNγ in non treated groups including; control sedentary rats (C), moderate trained (MT), overtrained (OT), and treated groups with** ***N. sativa*** **: control sedentary +** ***N. sativa*** **(NC), moderate trained +** ***N. sativa*** **(NM), overtrained +** ***N. sativa*** **(NO), (for each group, n = 6).** *; *P*< 0.05, compared to control group. ^##^; *P*< 0.01, comparison between overtrained and moderate exercise.

Serum level of IFNγ was significantly increased immediately after the last bout of exercise only in MT group (*P*< 0.05) compared with the control group. In addition, IFNγ concentration was significantly lower in the OT group than the MT group in both time periods (*P*< 0.01) (Figure 5).

**Figure 6:**
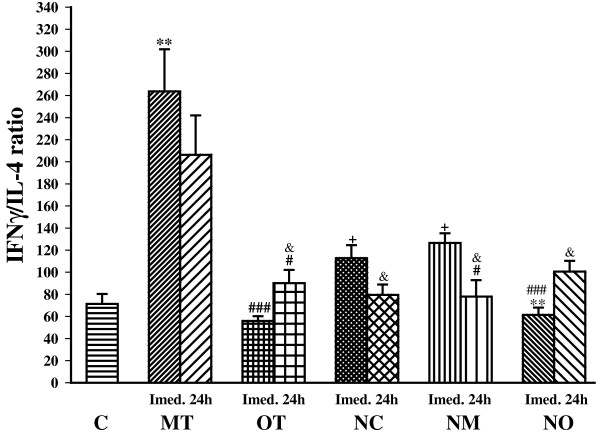
**The ratio of serum INF-γ/IL-4 (Th1/Th2 balance) in non treated groups including; control sedentary rats (C), moderate trained (MT), overtrained (OT), and treated groups with** ***N. sativa*** **: control sedentary +** ***N. sativa*** **(NC), moderate trained +** ***N. sativa*** **(NM), overtrained +** ***N. sativa*** **(NO), (for each group, n = 6).** **; *P*< 0.01, compared to control group. ^#^; *P*< 0.05, ^###^; *P*< 0.001, comparison between overtrained and moderate exercise.^+^; *P*< 0.05, comparison between treated and non treated groups. ^&^; *P*< 0.05, comparison between immediately or 24 h after last bout of exercise.

Moderate exercise (MT) increased IFN-γ/IL4 ratio (Th1/Th2 balance) compared with control (*P*< 0.001). However, there was no significant difference in IFN-γ/IL4 ratio between OT and control group. IFN-γ/IL4 ratio was significantly lower in the OT group compared with the MT group immediately (*P*< 0.001) and 24 h (*P*< 0.05) after exercise. Twenty four hours after the last bouts of exercise IFN-γ/IL4 ratio increased significantly in overtrained animals (*P*< 0.05) compared with immediately after the last bouts of exercise (Figure 6).

### The effect of exercise intensity on pro/anti-inflammatory cytokine ratio

**Figure 7:**
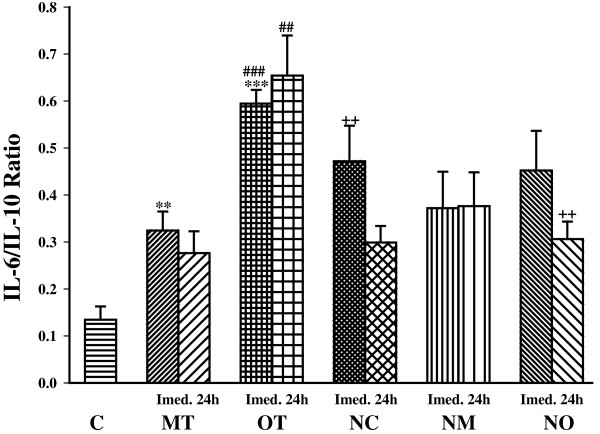
**The ratio of serum IL-6//IL-10 in non treated groups including; control sedentary rats (C), moderate trained (MT), overtrained (OT), and treated groups with** ***N. sativa*** **: control sedentary +** ***N. sativa*** **(NC), moderate trained +** ***N. sativa*** **(NM), overtrained +** ***N. sativa*** **(NO), (for each group, n = 6).** **; *P*< 0.01, ***; *P*< 0.001 compared to control group. ^##^; *P*< 0.01, ^###^; *P*< 0.001, comparison between overtrained and moderate exercise.^++^; *P*< 0.01, comparison between treated and non treated groups.

We measured IL-6/IL-10 and TNFα/IL-10 ratio to evaluate the pro/anti-inflammatory cytokine balance. Moderate exercise and overtraining significantly increased the IL-6/IL-10 ratio compared with control (*P*< 0.01 for MT and *P*< 0.001 for OT). IL-6/IL-10 ratio was also significantly higher in OT than MT group immediately (*P*< 0.001) and 24 h (*P*< 0.01) after exercise (Figure 7).

**Figure 8:**
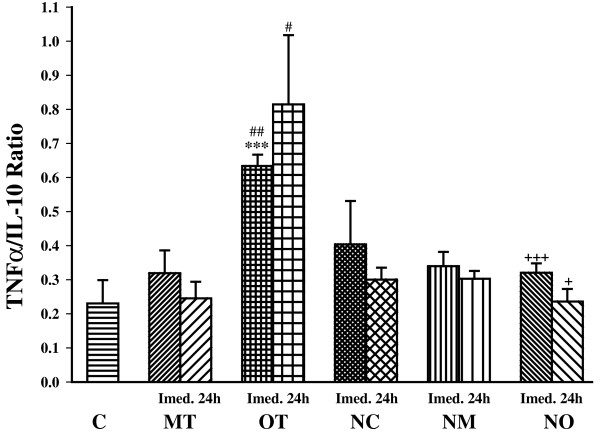
**The ratio of serum TNFα/IL-10 in non treated groups including; control sedentary rats (C), moderate trained (MT), overtrained (OT), and treated groups with** ***N. sativa*** **: control sedentary +** ***N. sativa*** **(NC), moderate trained +** ***N. sativa*** **(NM), overtrained +** ***N. sativa*** **(NO), (for each group, n = 6).** ***; *P*< 0.001, compared to control group. ^#^; *P*< 0.05, ^###^; *P*< 0.001, comparison between overtrained and moderate exercise.^+^; *P*< 0.05, ^+++^; *P*< 0.001, comparison between treated and non treated groups.

The ratio of TNFα/IL-10 was also significantly increased in OT group compared with control (*P*< 0.001) but its changes was not significant in MT group. In addition overtraining induced significant elevation in TNFα/IL-10 ratio compared with moderate exercise immediately (*P*< 0.01) and 24 h (*P*< 0.05) after exercise (Figure 8).

### The effect of *N. sativa*treatment on serum concentration of IL-6, TNFα, IL-10, IL-4 and IFNγ

*N. sativa* treatment caused a significantly higher level of IL-6 in NC and NM compared to control and MT groups (*P*< 0.01 for NC and *P*< 0.05 for NM). However, there was no significant difference in IL-6 concentration between OT and NO groups. There was not any significant difference between IL-6 concentrations in *N. sativa* treated groups (Figure 1).

*N. sativa* treatment increased TNFα in NC compared with the control (*P*< 0.01) immediately after the last bout of exercise, and in NM compared with MT group immediately and 24 h after the last bout of exercise (*P*< 0.01 for both times). However, *N. sativa* treatment decreased TNFα concentration in overtrained exercise animals (*P*< 0.05). There was not any significant difference between TNFα concentrations in *N. sativa* treated groups (Figure 2).

*N. sativa* treatment increased IL-10 concentration in control and exercised groups. It increased IL-10 concentration immediately after the end of the study in the NC group (*P*< 0.01) compared with the control. The IL-10 concentration increased in NM compared with the MT group immediately and 24 h (*P*< 0.01 for both times) after the last bout of exercise. In addition, *N. sativa* treatment increased IL-10 concentration in overtrained exercise animals immediately (*P*< 0.05) and 24 h (*P*< 0.001) after the last bout of exercise. In animals treated with *N. sativa*, there was not any significant difference in IL-10 concentration between groups. There were only statistical differences between immediately and 24 h IL-10 values in OT group (*P*< 0.01) (Figure 3).

*N. sativa* treatment significantly decreased IL-4 concentration in the control group (*P*< 0.05). It also increased IL-4 concentration in the NM group compared with the MT group immediately and 24 h after the last bout of exercise (*P*< 0.01 for both times). However, there was no significant difference in IL-4 concentration between OT and NO groups. In animals treated with *N. sativa*, there was not any significant difference between groups. After 24 h IL-4 values decreased in OT and NO groups (*P*< 0.01 for both), but it increased in NM groups (*P*< 0.05) compared to immediately after the last bouts of exercise (Figure 4).

There was no significant difference in IFNγ concentration between treated and non treated groups. In animals treated with *N. sativa*, there was not any significant difference between groups (Figure 5).

### The effect of *N. sativa*treatment on Th1/Th2 balance

*N. sativa* treatment increased this ratio in the control group (NC) while decreased it in the moderate training (NM) animals compared with non treated animal immediately after exercise (*P*< 0.05 for both groups) and 24 h after exercise for NM group (*P*< 0.05). In addition, the IFN-γ/IL4 ratio in the NO group was lower than the NC and the NM groups immediately after exercise (*P*< 0.01 for NC and *P*< 0.001 for NM). Twenty four hours after the last bouts of exercise IFN-γ/IL4 ratio increased significantly in overtrained animals (NO groups) but decreased significantly in the control and the moderate trained animals treated with *N. sativa* (*P*< 0.05 for all groups) compared with immediately after the last bouts of exercise (Figure 6).

### The effect of *N. sativa*treatment on pro/anti-inflammatory cytokine ratio

*N. sativa* treatment increased IL-6/IL-10 ratio in the control group (NC) (*P*< 0.01), while it didn’t significantly changed the IL-6/IL-10 ratio in moderate trained animal. In addition IL-6/IL-10 ratio non significantly decreased in overtrained treated animal (NO) immediately after exercise but it was significantly decreased compared with overtrained animal 24 h after exercise (*P*< 0.01). In animals treated with *N. sativa*, there was not any significant difference between groups (Figure 7).

*N. sativa* treatment significantly decreased TNFα/IL-10 ratio in overtrained animal (NO) immediately (*P*< 0.001) and 24 h (*P*< 0.05) after exercise but there was not significant differences between other treated and non treated animals. In animals treated with *N. sativa*, there was not any significant difference between groups (Figure 8).

## Discussion

To our knowledge, this is the first animal study to investigate the effects of *N. sativa* treatment, in prolonged overtraining endurance treadmill exercise and moderate training on cytokine profile and the immune system.

In untreated animals there was a chronic increase in pro-inflammatory cytokine TNFα and inflammation responsive cytokine IL-6 in OT group, which remained elevated after 24 h recovery. The concentration of anti-inflammatory cytokines IL-10 also increased significantly immediately after overtraining but this change was acute and not lasted for 24 h. We showed a significant elevation in IL-6/IL-10 ratio (for both exercise groups) and TNFα/IL-10 ratio (for just overtraining group) which may indicate the load dependent effect of exercise on pro/anti-inflammatory cytokine balance. In addition, this load of overtraining caused no change in acute IL- 4 (Th2 cytokine) and IFNγ (Th1 cytokine) concentration. However, after moderate training significant increase in IL-6 and IFNγ concentration and decrease in IL-4 concentration was observed. The marked post overtraining exercise increase in IL-6 and IL-10 concentration and pro/anti-inflammatory cytokine ratio changes have been confirmed by previous studies [23, 29–32]. A chronic increase in pro-inflammatory cytokine TNFα and inflammation responsive cytokine IL-6 which remained elevated at least two weeks after recovery had been shown in our previous study [23]. Speaker et al showed that regular exercise may ameliorate stress-induced enhancement of innate immune function through increases in stress-evoked Hsp72, MCP-1, IL-6, and IL-10 and decreases in IL-1β/IL10 and TNF-α/IL10 ratios within white adipose tissue [33]. Contracting skeletal muscle is the main source of IL-6 in the circulation in response to exercise [34]. Exercise induces IL-6 mRNA up regulation [35–37] and it’s gen transcription [38] in contracting skeletal muscle was shown. In addition muscle damage can cause mild inflammation and infiltration of macrophages and neutrophils into damaged tissue in 6-48 hr after exercise, which in accompany with adipose tissue may increase exercise-induced augmentation of IL-6 at recovery time [39]. IL-6 has a metabolic role by signaling the liver to increase glucose output and prevent severe drop in glucose concentration in the exercise and it may has a lipolytic role [36, 40–43]. In Lira et al. study, overtraining increases the concentration of IL-6 and IL-10 in adipose tissue and activates NFκB p65 and TLR4 pathways. Our results also confirmed this hypothesis that TNFα may stimulate the production of IL-6 which is in turn stimulates the production of IL-10 and cortisol [18, 44].

In addition, as we showed in previous and this study, the Th1 responses are strengthened following moderate intensity exercises and Th2 after overtraining [20, 23]. Tissue trauma after overtraining would activate local cells to produce cytokines stimulating the differentiation of Th2 cells. In addition post overtraining stress hormones (cortisol and catecholamines) elevation can inhibit the production of IL-12 (main inducer of Th1 cells) and would up-regulate Th2 lymphocyte responses [45–47].

Despite existing many recommendations to use *N. sativa* as tonic and adaptogenic herb, little information is available about its tonic properties in exercises. Therefore, the immunomodulatory and tonic effects of *N. sativa* were examined by oral administration of its ethanolic extract in control, moderate and overtrained exercises. In treated control (NC) group IL-6, TNFα and IL-10 concentrations significantly increased, but IL-4 significantly decreased and there were no significant changes in IFNγ values, so IFNγ/ Il-4 ratio increased significantly compared to non treated control. Although, the pro-inflammatory cytokines such as IL-6 and TNFα increased in supplemented sedentary group, the anti-inflammatory cytokine such as IL-10 was also increased in this group. Therefore INF-γ/IL-4 increment and no change in TNFα/IL-10 ratio in this group may clearly indicate that *N. sativa* supplementation did not cause inflammation.

In NM group IL-6, TNFα, ΙL-10 and IL-4 concentrations increased significantly, but the value of IFNγ didn’t change and the IFNγ/ Il-4 ratio decreased significantly in comparison to non treated moderate exercise group. In NO group TNFα concentration decreased and IL-10 increased significantly but the values of IL-6, IL-4, IFNγ and the IFNγ/ Il-4 ratio didn’t change in comparison to non treated overtraining group; which showed more pronounced anti-inflammatory effect of *N. sativa* treatment in this group. The decreased in pro/anti-inflammatory cytokine ratio (IL-6/IL-10 and TNFα/IL-10) in overtrained treated animal may confirmed this immunomodulatory effect too.

Considering the chronic effects of *N. sativa* treatments (values after 24 h) and the lack of difference between treated groups’ cytokines, the following hypothesis may be proposed. *N. sativa* may change the set point of the immune system and stimulate both pro and anti-inflammatory cytokines. Also it may act as a balancing factor on Th1/Th2 lymphocytes in different exercise load and an inhibitory factor on Th2 phenotype in the control group.

In all treated groups of this study, IL-10 concentrations increased but, IL-4 (both cytokines are secreted from Th1 cells) values changes were different in various groups. Indicating that, IL-10 may be secreted by other cells (adipocytes) than immune cells [26]. The pattern of IL-6/IL-10, TNFα/IL-10 and IFNγ/IL-4 changes in treated groups indicated that *N. sativa* ethanolic extract effects were highly specific to cytokine involved and immune system status. The results showed that its anti-inflammatory effects were more pronounced in overtraining animals than control or moderate exercise animals. In fact, *N. sativa* supplementation caused an Immunoregulatory effect which somehow homogenizes immune state among different groups. The present study results also showed that *N. sativa* ethanolic extract may have immuno-stimulatory and immune-suppressive components. Previous studies had shown bias finding about the effect of *N. sativa* on cytokines profile. In a recent study *N. sativa* had increased both the IL-10 and TNFα concentration in normal rats, but in another study thymoquinone (*N. sativa’s* major bioactive component) reduced TNFα and increased IL-10 in the rat’s arthritis model [48, 49].

The effect of oral administration of the extract of *N. sativa* on IL-4, IFNγ and the IFNγ/ Il-4 ratio (Th1/Th2 balance) on sensitized animals have been shown previously. While both IL-4 and IFNγ were increased in sensitized guinea pigs, treatment with *N. sativa* resulted in further increase in IFNγ but reduction of IL-4 and therefore increased IFNγ/ Il-4 ratio. Treatment of sensitized animals with the extract also leads to reduction of pathological changes of the lung [8]. The main constituent of *N. sativa,* thymoquinone also showed similar results [4].These results support the findings of the present study and a balancing role of *N. sativa* on Th1/Th2 lymphocytes.

According to Haq et al reports, aqueous extract of *N. sativa* enhanced TNFα but not IL-4 production in non-activated, mitogen activated or allogeneic cells, and stimulate IL-3 production by T cells [50, 51]. Therefore, *N. sativa* may have a stimulatory effect on macrophage directly or indirectly through IL-3 [50].

Although, previous studies showed that lipid - soluble component such as thymoquinone is mainly responsible for the anti-inflammatory effect of the plant [1, 4, 48, 52] it is not obvious that water solvable (such as proteins) or lipid- soluble (such as thymoquinone) constituents of the plant are responsible for its effects [53]. Therefore, the hydroethanolic extract of the plant was used to extract the both lipid and water soluble constituents of *N. sativa*. However, more investigation should be done to evaluate the safety of long-term administration of herbal medicine and their constituents.

## Conclusion

In conclusion, chronic administration of *N. sativa* may change pro and anti-inflammatory cytokines profiles. Also it may act as a balancing factor on Th1/Th2 lymphocytes in different exercise loads and act as an inhibitory factor on Th2 phenotype in control animals.
